# IGEG-2 is a *C. elegans* EGFR ligand

**DOI:** 10.17912/micropub.biology.001821

**Published:** 2025-09-15

**Authors:** Melissa M Mailhot, Jesse G Jones, Dylan L Castro, Cheryl Van Buskirk

**Affiliations:** 1 Department of Biology, California State University, Northridge, Northridge, California, United States

## Abstract

Across species, Epidermal Growth Factor (EGF) family ligands and their receptors participate in developmental and physiological cell-cell signaling events. *C. elegans* possesses a single EGF receptor, LET-23 /EGFR, and two characterized EGF ligands. LIN-3 /EGF is well-known for its role in vulval induction, and SISS-1 /EGF mediates stress-induced sleep. The *C. elegans* genome harbors another predicted EGF family member, *igeg-2 ,* which has not been characterized. To determine if IGEG-2 is a functional EGFR ligand, we examined whether it can activate known LET-23-dependent processes. We found that ubiquitous overexpression of IGEG- *2* promotes both vulval induction and sleep, indicating that it is a functional EGF family ligand. The endogenous role of IGEG-2 remains unknown.

**Figure 1. Widespread overexpression of IGEG-2 promotes EGFR-dependent events:**
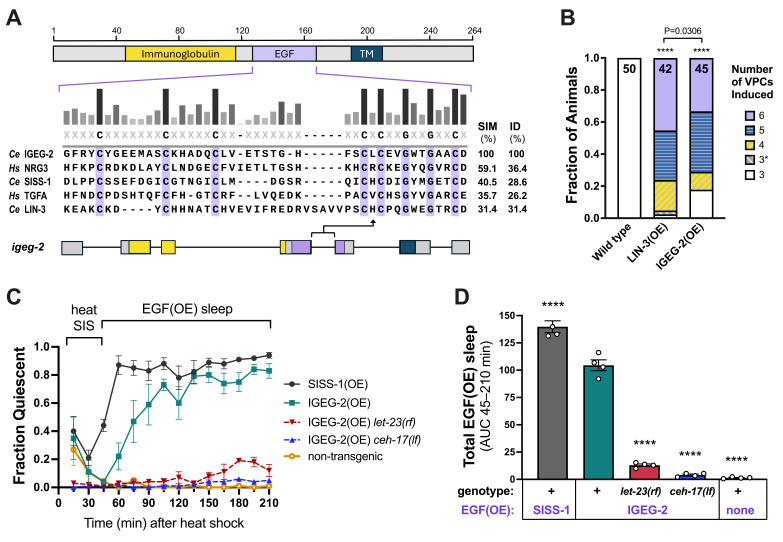
**A)** Predicted domain structure of IGEG-2 (top), EGF domain alignments (middle), and *igeg-2* intron-exon structure (bottom). The IGEG-2 protein contains a predicted extracellular immunoglobulin domain (residues 46-117), an EGF domain (127-168), and a transmembrane domain (188-210) (UniProt Consortium 2025). Multiple sequence alignment of the EGF domains of IGEG-2 and selected EGFR ligands is shown in order of pairwise similarity to IGEG-2 . NRG3 = human Neuregulin 3, TGFA = human TGF alpha. The *igeg-2* gene contains an intron between the 4 ^th^ and 5 ^th^ cysteines of the EGF domain, a highly conserved feature among EGF homologs. **B** ) Number of vulval precursor cells (VPCs) induced to adopt vulval fates following overexpression of LIN-3 or IGEG-2 during the late L2 stage. In wild-type animals, three out of six VPCs are induced, with P6.p adopting a primary fate and P5.p and P7.p adopting secondary fates. In EGF(OE) animals, up to six VPCs adopt vulval fates. 3* represents animals with excess of primary fates among P5.p-P7.p, with no additional VPCs induced. The number of animals examined is indicated at the top of each bar. ****P<0.0001 vs. wild type, Fisher's exact tests of categorical data, 3 VPCs versus hyperinduced (all other categories combined). **C** ) Fraction of animals showing behavioral quiescence (sleep) over time following transgene induction at the young adult stage. Heat SIS refers to endogenous heat stress-induced sleep, while EGF(OE) sleep refers to overexpression-dependent sleep. **D** ) Area under the curve (AUC) of EGF-induced quiescence (45-210 min) from panel C. + = wild type, rf = reduction of function, lf = loss of function, OE = overexpression. Each data point represents four trials of 25 animals, assayed with experimenter blind to genotype. ****P<0.0001, one-way ANOVA with Dunnett's multiple comparisons test vs. IGEG-2 (OE). The greater total sleep of SISS-1 (OE) relative to IGEG-2 (OE) is partly due to a difference in sleep onset: P=0.0055 AUC 45-90 min, P=0.0990 AUC 105-210 min, two-tailed unpaired t-test SISS-1 (OE) vs. IGEG-2 (OE). All statistical analyses were performed using GraphPad Prism software.

## Description

The nematode *C. elegans* possesses a single EGF receptor encoded by *let-23* (Aroian et al. 1990; Stein & Staros 2006), and two EGF family ligands encoded by *lin-3* (Hill et al. 1992) and *siss-1* (Hill et al. 2024). LIN-3 – LET-23 signaling impacts viability, fertility, vulval induction, P12 neuroblast specification and male spicule development (Aroian & Sternberg 1991), whereas SISS-1 – LET-23 signaling promotes sleep following tissue damage (Hill et al. 2024). Both LIN-3 and SISS-1 were identified in genetic screens for defects in EGFR-dependent processes (Hill et al. 1992; Hill et al. 2024). EGF family ligands are also identifiable bioinformatically as transmembrane proteins with an extracellular EGF domain: a receptor-interaction motif containing six cysteines with conserved spacing (Harris et al. 2003). Indeed, SISS-1 ( **s** tress- **i** nduced **s** leeples **s)** had originally been assigned the name IGEG-1 for its **I** mmuno **g** lobulin-like and **EGF** domains, and a second gene with a similar domain structure had been assigned the name IGEG-2 ( Fig. 1A ) (Sternberg et al. 2024). LIN-3 , SISS-1 , and IGEG-2 are not likely to have arisen by recent gene duplications, as their EGF domains are no more closely related to each other than to those found in other taxa ( Fig. 1A ). Another near-invariant feature of EGF family ligands is the presence of an intron between the closely spaced 4th and 5th cysteines of the EGF motif (Stein & Staros 2006), and this intron placement is found in *igeg-2* ( Fig. 1A ).

To investigate whether IGEG-2 is a functional EGFR ligand, we first examined whether it can promote LET-23 /EGFR signaling within the vulval epithelium. Vulval organogenesis is normally mediated by LIN-3 released from the gonadal anchor cell of the late L2 larva, activating LET-23 within three of six multipotent vulval precursor cells (VPCs) (Wang & Sternberg 2001). LIN-3 overexpression at the L2 stage from a heat-responsive promoter leads to hyperinduction, with up to six VPCs adopting vulval cell fates (Katz et al. 1995). We placed a full-length *igeg-2* cDNA under the same heat-responsive promoter and examined whether IGEG-2 overexpression promotes vulval hyperinduction. We found that IGEG-2 (OE) at the L2 stage results in up to six VPCs adopting vulval fates ( Fig. 1B ), suggesting that IGEG-2 functions as an EGFR ligand.

We then examined whether IGEG-2 (OE) at the adult stage can trigger LET-23 /EGFR activation within sleep-promoting neurons. Following tissue damage, SISS-1 released by damaged cells promotes stress-induced sleep (SIS) via LET-23 activation within the peptidergic ALA and RIS neurons (Hill et al. 2024; Konietzka et al. 2020). Overexpression of either LIN-3 or SISS-1 drives a prolonged and robust bout of EGFR-dependent sleep (Van Buskirk & Sternberg 2007; Hill et al. 2024), and we wished to determine whether overexpression of IGEG-2 does the same. We found that IGEG-2 overexpression drives behavioral quiescence similar to SISS-1 (OE) but with a slightly delayed onset ( Fig. 1C, D). We then wished to examine whether this IGEG-2 (OE) sleep is EGFR-dependent. Given that complete loss of LET-23 is associated with lethality (Herman 1978), we examined the partial reduction of function allele *let-23 ( n1045 ),* which is cold-sensitive (Ferguson & Horvitz 1985). We found IGEG-2 (OE) sleep to be nearly abolished in *let-23 ( n1045 )* mutants grown at the non-permissive temperature ( Fig. 1C, D). Further, we found that IGEG-2 (OE) sleep is abolished in *ceh-17* null mutants ( Fig. 1C, D), which are impaired for LET-23 expression within the sleep-promoting ALA neuron (Van Buskirk & Sternberg 2010). These data support the notion that IGEG-2 , at least when overexpressed, promotes EGFR activation.

With the identification of IGEG-2 /EGF, *C. elegans* appears to have at least three EGFR ligands. LIN-3 mediates several signaling events including those that impact viability and vulval induction (Liu et al. 1999), SISS-1 mediates stress-induced sleep following tissue damage (Hill et al. 2024), and the role of IGEG-2 is not yet apparent, as *igeg-2* null mutants are superficially wild type. Ubiquitous overexpression of any of these ligands can promote sleep via activation of LET-23/EGFR within sleep-promoting neurons. The onset of IGEG-2 (OE) sleep is delayed relative to SISS-1 (OE), pointing to rate-limiting differences in post-transcriptional regulation. Overexpression of LIN-3 or IGEG-2 at the L2 stage promotes hyperinduction of vulval cell fates, while SISS-1 (OE) does so only very weakly (Hill et al. 2024). These data suggest that SISS-1 may be a lower-affinity ligand than the others, and/or that its ability to interact with LET-23 /EGFR is restricted in a context-dependent manner. Like SISS-1 , the ectodomain of IGEG-2 contains an immunoglobulin-like domain, though the Ig domains of these two ligands are not similar in sequence (UniProt Consortium 2025). The function of the Ig domain within Ig-EGFs, such as *Drosophila* Vein and certain human Neuregulins, is not well understood. Characterization of the function of IGEG-2 and its structural requirements may contribute to our understanding of Ig-EGFs across species.

## Methods

**EGF domain alignment:** *C. elegans* and human EGF domains were identified based on UniProt annotations derived from PROSITE-ProRule PRU00076 (Sigrist et al. 2005; Uniprot Consortium 2025). EGF domains from four residues upstream of the first conserved cysteine to one residue after the sixth conserved cysteine were subjected to pairwise sequence comparison with IGEG-2 using the ebi.ac.uk EMBOSS NEEDLE tool (Madeira et al. 2022) to derive percent identity and similarity values. EMBOSS Needle parameters are as follows: matrix=BLOSSUM62, gap open=10, gap extend=0.5, END GAP=false, end gap open=10, end gap extend=0.5. Multiple sequence alignment of the EGF domains of selected EGFR ligands were generated with the MUSCLE alignment tool (default settings, version 3.8.1551) within SnapGene software (www.snapgene.com).

**Transgene construction:** The hs: LIN-3 (Van Buskirk & Sternberg 2007) and hs: SISS-1 (Hill et al. 2024) transgenes have been previously described. The hs: IGEG-2 transgene was constructed in a similar manner by cloning the *igeg-2* cDNA (transcript F48C5.1.1) into pPD49.83 (Addgene plasmid #1448). The *igeg-2* cDNA included 15 bp of 5'UTR and 140 bp of 3'UTR and was synthesized by Twist Biosciences with added 5' KpnI and 3' SacI sites. This 962 bp fragment was cloned KpnI-SacI into pPD49.83, placing *igeg-2* under control of the *hsp-16.41* promoter and adding 3'UTR from the *unc-54* gene. The resulting plasmid pCV50 was injected at 10 ng/ul along with the coinjection marker *col-12* p:dsRED into N2 . Three lines carrying independent extrachromosomal transgenic arrays were examined for IGEG-2 (OE) sleep and a representative transgene, csnEx7 , was used in this study.

**EGF(OE) sleep:** Transgenic hs:EGF and non-transgenic siblings were placed on 35 × 10 mm (5 ml) NGM plates seeded with OP50 *.* The plates were sealed with parafilm and heat-shocked at 35°C in a water bath for 20 minutes to induce transgene expression. Plates were then cooled on frozen LabArmor beads for 1 minute, parafilm was removed, and 25 animals per genotype were examined at each time point under a stereomicroscope for behavioral quiescence indicative of sleep. Animals that were immobile and did not show pharyngeal pumping over a three-second observation were scored as quiescent.

**Vulval induction:** PS5970 , CVB82 and N2 control animals were heat-shocked as above but at 33 °C for 30 minutes at the late L2 stage and examined at the L4 stage by DIC microscopy (Zeiss Axio Imager A2) for ventral epithelial invaginations indicating VPC induction (Wang & Sternberg 2001).

## Reagents

**Table:** 

**Strain name**	**Genotype**	**Source**
N2	wild type	CGC
PS5970	*syIs197* [ *hsp-16.41* p:pro- *lin-3* c, *myo-2* p:dsRED]	CGC
CVB56	*csnEx1* [ *hsp-16.41* p:pro- *siss-1* b, *myo-2* p:GFP]	CVB
CVB82	*csnEx7* [ *hsp-16.41* p:pro- *igeg-2* , *col-12* p:dsRED]	CVB
CVB118	*csnEx7* ; *ceh-17* ( *np1* )	CVB
CVB119	*csnEx7* ; + (wild-type isolate from *ceh-17* outcross)	CVB
CVB120	*csnEx7* ; *let-23* ( *n1045* )	CVB

CGC = Caenorhabditis Genetics Center, CVB = Van Buskirk lab. *let-23* ( *n1045* ) is a cold-sensitive reduction-of-function allele (Ferguson & Horvitz 1985) and *ceh-17* ( *np1* ) is a molecular null allele (Pujol et al. 2000). CVB118 and CVB119 were generated by crossing CVB82 with IB16 *ceh-17* ( *np1* ), with CVB119 serving as the wild-type IGEG-2 (OE) control for sleep experiments. CVB82 and CVB119 behave similarly. All strains were fed OP50 and grown at 15˚C, the non-permissive temperature for the *let-23* ( *n1045* ) allele.
